# Preface to the proceedings of Halophiles 2013

**DOI:** 10.3389/fmicb.2015.00341

**Published:** 2015-04-22

**Authors:** R. Thane Papke

**Affiliations:** Department of Molecular and Cell Biology, University of ConnecticutStorrs, CT, USA

**Keywords:** *Haloferax volcanii*, halophilic and halotolerant microorganisms, halophile biochemistry, halophile molecular biology, halophile metabolism, halophile adaptations, halophile communities, halophile evolution

## Introduction

Halophiles are organisms, and their viruses, adapted to thriving and reproducing in high salt concentrations, representatives of which can be found in all domains of life. To flourish in these environments, an individual must overcome many obstacles including high osmotic pressure, low water availability, high and low pH conditions, high temperatures, and high cell densities that enforce stiff competition for limited resources due to the low solubility of gasses and other nutrients. Therefore, all halophiles can be considered poly-extremophiles.

These interesting life forms do not cause infectious diseases or cancer, impact human lifestyles or infect our food supply. So why study them? Often halophiles are studied because the salty environment is a model system for uncovering basic principles of microbial life. This extreme environment selects against any organism not able to cope with high salt, which reduces the overall community structure and function. The expectation is that this decrease in complexity allows for more easily achieved insights into fundamental microbial adaptations, and ecological, biogeochemical and evolutionary processes. Because the extreme hypersaline environments are limited to only microbial life, the habitat is analogous to that which existed on Earth before the Cambrian Explosion. Thus investigations of hypersaline habitats can deliver insight into the longest epoch of life. Modern microbial mats growing in hypersaline habitats are similar in structure to ancient stromatolites found in the Precambrian fossil record. Related to this is the search for life in the cosmos: the long period of dominant microbial life on Earth suggests a higher likelihood of finding microbial life on another world than finding advanced life, sentient beings, or even trees. Additionally, the adaptations required for life in high salt can produce enzymes that are interesting for biotechnology, industrial processes, and bioremediation.

Every 3 years since the late 1970s halophile researchers who focus on microbial life in hypersaline environments have gathered to present their latest exciting research to like-minded souls. This eBook is a compendium of research written by many of the presenters at Halophiles 2013, the international congress held at the University of Connecticut in Storrs, CT (to view all of the conference oral and poster presentation abstracts, please visit https://www.regonline.com/custImages/250000/250066/Halophiles2013Program_final.pdf). Its range of subject matter is extensive reflecting the 4-day event, and the breadth of research interests. To address this diversity of topics, the chapters are arranged into slightly narrower areas of research interests, but even within these defined partitions the reader will find a wide range of interesting research. Here, we explore the world of halophiles.

## A league of their own

The lead article by Baxter et al. (2014) is based on the keynote talk of the conference, which is a reflection and commentary on gender bias in science and more specifically in the field of halophiles. The authors conducted a thorough study of women's success in halophile research through time by measuring who gives invited lectures at the Halophile conferences, an honor that connotes peer recognition obtained through individual hard work and dedication. The authors found that the field of halophiles is “an example of progress” achieving gender equitability not found in many fields. Further, many significant women researchers and their contributions to the field are highlighted in this paper: it is a long, and compelling list.

## Communities, diversity, and evolution

The first chapter in the section on Communities, Diversity, and Evolution is the most frequently viewed article from the conference proceedings; it has been viewed several 100 times more often than its closest competitor, and more than a 1000 compared to the 3rd most viewed contribution. There is no surprise it was written by Mormile (2014), one of the featured women in the Baxter et al., study. This manuscript highlights the metabolism and adaptations discovered through sequencing the *Halanaerobium hydrogeniformans* genome, a hydrogen producing haloalkaliphile cultivated from Soap Lake, WA.

The second chapter in this section by Fathepure (2014) is a review on the bioremediation of petroleum compounds in hypersaline environments. Oil production generates hypersaline waters due to the extraction process, and many petroleum rich regions are surrounded by natural hypersaline systems like sabkhas or coastal salt marshes that experience crude oil pollution. Therefore, remediation of “production water” and polluted natural brines requires for decontamination microbes and enzymes that are adapted to function at elevated NaCl concentrations. This manuscript gives an up to date review on the state of the field.

Grotzinger et al. (2014) used cell sorting followed by whole genome amplification from single cells and genome sequencing to examine communities that live in hypersaline pools existing at the bottom of the Red Sea. They were particularly interested in discovering genes from halophiles that might be of commercial value (e.g., hydrolases, dehydrogenases). To aid their cultivation independent search, they developed a bioinformatic tool called a profile and pattern matching algorithm to find genes of interest. In this manuscript they detail their efforts, successes and failures, at mining the single amplified genome data.

Fullmer et al. (2014) sequenced genomes from 19 *Halorubrum* strains all cultivated from the hypersaline lake Aran Bidgol located in Iran and compared them to *Halorubrum* genomes available at the NCBI database. Multilocus sequence analysis was used to establish a phylogeny among strains and the robustness phylogenetic clusters was tested by average nucleotide identity and G + C content, which showed conformity unless groups were comprised of strains cultivated from different geographic locations. Inteins and clustered regularly interspaced short palindromic repeats (CRISPRs) within and between phylogenetic assemblages were patchily distributed among all strains including those that were >99.5% identical for DNA sequence across five protein coding loci.

In the next chapter, Dillon et al. (2013) examined different salinity ponds from the Guerrero Negro solar saltern in Baja California Sur, Mexico using 16S rRNA and bacteriorhodopsin genes as molecular markers for assessing microbial diversity. This study showed that contrary to expectations, ponds of similar salinity had variable community structure; bacterial exceeded archaeal diversity; one of the ponds was dominated by clones that were unrelated to *Salinibacter ruber*, the typically reported bacterial inhabitant of saturated brines. The authors also showed that the haloarchaeal diversity in lower salinities was largely previously uncharacterized.

Multilocus sequence analysis and genome fingerprinting of 43 *Halorubrum* and *Haloarcula* strains cultivated from the hypersaline lake Aran Bidgol in Iran (Mohan et al., 2014) showed that these haloarchaeal genomes were exceedingly dynamic: nearly every strain examined had a unique fingerprint, even ones with 100% DNA sequence identity for five protein coding genes (~2500 nt). As a result, the authors concluded that the accumulation of this dramatic genomic variance was due to extensive gene gain and loss, which occurred faster than the neutral mutation rate.

Inteins are selfish genetic elements that insert themselves into highly conserved proteins. Through extensive haloarchaeal genome analysis (118 genomes, from 26 genera) Soucy et al. (2014) showed that most inteins had invaded only a fraction of the available insertion sites, though some were more adept than others. The absence of inteins despite available invasion sites indicates an inability to be mobilized likely based on the capacity of cells to exchange DNA with other community members. Therefore, the authors suggested gene transfer is not random in the haloarchaea, but instead exhibits extensive biases.

The study by Fernandez et al. (2014) used metagenomics to compare the community structure and functional properties between two saltern systems located on different coasts of Spain. Of significance was the observation that middle salinity concentrator ponds can have highly variable communities. The community from the Atlantic saltern 21% NaCl pond was more similar in structure to the Mediterranean saltern 33% pond, rather than a similar salinity pond (19%) at the same location. Additional analyses indicated carbon and phosphate availability might have a greater effect than NaCl concentrations in determining community structure.

Raman spectroscopy has become a useful tool in assessing biomolecules and minerals in strains and communities. Here, Jehlicka and Oren (2013) review how this technique has been applied to halophile communities living in gypsum crusts, evaporitic sediments, halite inclusions and endoliths, as well as to cultures for the purpose of describing the detection and distribution of important microbiological and geochemical markers.

Sencilo and Roine (2014) review what is known about the genomes of tailed viruses that infect haloarchaeal cells. Perhaps it is not surprising that these viruses are highly adapted to their hosts as their DNA G + C percentage is very high, like that of their hosts. Unexpectedly however, it appears they have much in common with their bacterial counterparts including genome content and organization, and similar capsid architecture and assembly. Thus, these strong commonalities suggest deep evolutionary relationships and a possible common ancestor for all tailed viruses/phage.

Nazareth and Gonsalves (2014) cultivated halophilic *Aspergillus* strains from many different hypersaline environments and characterized their ability to grow in different concentrations of salt, and on different carbon sources. Conidia germination and morphological changes in response to different salt concentrations were also examined. As no growth or conidia germination was detected in media without salt, and optimum growth was determined to be around 10%, these fungal strains were considered truly halophilic.

## Adaptations and metabolism

Despite being available in all habitats, little is known about DNA as a nutrient. In this publication by Chimileski et al. (2014) *Haloferax volcanii* was used as a model organism for exploring extracellular DNA metabolism. *Hfx. volcanii* grew best on DNA as a phosphate source, only slightly as a nitrogen source, and not at all as a carbon source. Furthermore, these cells were fussy about the sources of DNA they consumed and the bias was based on DNA methylation. These authors also identified and confirmed the gene HVO_1477 is required for growth on DNA, and that its homologs are widespread in archaea.

This review by Oren (2013) examines the dogma surrounding the linkage between excessive acidic amino acids in the proteomes of cells and the presence of high intracellular KCl concentrations used for osmotic balance. While the canonical examples *Halobacteriales* and *Salinibacter ruber* demonstrate both the salt-in strategy and an acidic proteome, recent genomic and metagenomic analyses revealed the decoupling of those two phenotypes. These new findings are unexplained but it is clear that our current understanding is too simplistic.

Plemenitas et al. (2014) review what is known about fungal adaptations to high salt concentrations from a molecular and genomic perspective, by comparing the salt tolerant *Hortaea werneckii* and the obligate halophile *Wallemia ichthyophaga*. They show that though signaling pathways necessary for sensing and responding to increasing salt concentrations are conserved between them, the observed structural and regulatory differences could account for their overall salt adaptations. Further, genomic analyses show substantial evolutionary or adaptation strategy differences between them.

The presence of multiple chromosome copies offers many advantages to the survival of cells that have this phenotype. Zerulla and Soppa (2014) review these advantages for species of haloarchaea, which have demonstrated high copy numbers, even in stationary phase. Most evolutionary explanations for the presence and origin of polyploidy are based on repair of damaged or mutated DNA and require the precondition of homologous recombination. Recent work on copy number and DNA as phosphate source however suggest polyploidy could stem from a need for intracellular phosphate storage.

Circadian rhythm has been studied in two of the three domains of life but nothing was known about the subject in Archaea, except for the notable presence of cyanobacterial *Kai-family* genes. Maniscalco et al. (2014) studied *cir* gene expression in *Haloferax volcanii* and demonstrated that those homologs are upregulated during 12 h diurnal cycles compared to dark conditions alone: they also showed that gene knockouts disrupted rhythmic gene expression. This groundbreaking work should cast bright light on archaeal circadian rhythms.

Metabolism of dihydroxyacetone (DHA) in the haloarchaea was thought only to occur in the species *Haloquadratum walsbyi*, and requires kinases. This manuscript by Ouellette et al. (2013) demonstrated *Haloferax volcanii* also grows on DHA as a sole carbon source, and that though phosphylated by a DHA kinase, phosphorylation of DHA primarily occurs by a glycerol kinase. Further, genomic analyses unexpectedly showed that DHA and glycerol kinases are widespread throughout the haloarchaea, suggesting DHA is an important nutrient for all species to metabolize.

In this review chapter by Sinha and Khare (2014), the role of salt in controlling the stability of proteins is explored. Though it has been known that proteins adapted to hypersaline conditions typically are not functional without salt, the presence of salt also provides proteins with protection against the denaturing effects of temperature, chaotropic agents, organic solvents, and mutations. Understanding how this effect works could lead to the development of better biocatalysts.

## Biochemistry and molecular biology

Talon et al. (2014) used malate dehydrogenases from *Chloroflexus aurantiacus* and *Salinibacter ruber* as models for understanding the adaptations required for the solvation of proteins under hypersaline conditions. They demonstrated that water molecules have indirect and direct hydrogen bonds with the *C. aurantiacus* and *S. ruber* proteins respectively, which stabilized the particular versions. The substitution of non-polar amino acids in *C. aurantiacus* by acidic ones on the surface of the *S. ruber* protein was noted and thermodynamic arguments indicated these were the appropriate adaptations to high internal salt concentration experienced by *S. ruber*, and by extension all haloarchaea.

The DNA replication helicase catalytic core is homologous between eukaryotes and archaea, suggesting an archaeal model organism can provide deeper understanding into its structure and function. In this chapter by Kristensen et al. (2014) the first extensive *in vivo* genetic manipulation of the MCM complex for an archaeaon is reported. Guided by multiple sequence alignments and a crystal structure many conserved amino acids –singletons, small clusters and larger clusters- were deleted and assessed for impact. Results indicate that *Haloferax volcanii* is an excellent model organism for reverse genetic analysis of MCM, and other key eukaryote homologs.

Yatsunami et al. (2014) report here the discovery, the cellular composition, and the antioxidant potential of carotenoids produced by the previously uninvestigated species *Haloarcula japonica*. Their results suggest that *H. japonica* may have very high carotenoid content compared to other haloarchaeal species, which may confer higher resistance to damaging radiation.

The origin and evolution of amino acids is largely inferred but a consensus of 10 are suggested to have been available in the prebiotic Earth. Here Longo and Blaber (2014) report the analysis of enriching a protein with the set of prebiotic amino acids and determining its folding potential. They noticed that proteins remained stable when the core was hydrophobic and the surface had a high negative charge (i.e., acidic amino acids). Both of these characteristics are found in proteins adapted to being soluble in high salt concentrations leading the authors to suggest the prebiotic early earth environment may have been very salty.

Compatible solutes are organic molecules intracellularly accumulated in many halophiles to balance the osmotic pressure of their external environment. Hydroxyectoine is a common compatible solute and it also protects cells and proteins from desiccation, and heat. Tanne et al. (2014) show that hydroxyectoine produced by *Chromohalobacter salexigens*, from the family *Halomonadaceae*, has physical properties that allows the biological processes of cells to continue functioning even in a dehydrated condition.

The moderate halophile *Halobacillus halophilus* produces glutamate and glutamine as compatible solutes. Shiyan et al. (2014) hypothesized that the annotated glutamine syntetase A2 found in the *H. halophilus* genome was key to the biosynthesis of glutamate and glutamine as a compatible solute. To their surprise, knock out analysis revealed this enzyme was not involved, indicating some unknown enzyme must be responsible for generating these compatible solutes.

The post translational addition of glycans to proteins (N-glycosylation), though found in all domains of life, is an important adaptation for organisms living in the high salt environment, as modification in response to changing salinity conditions provides flexibility to a protein's ability to remain soluble and functioning. This chapter by Eichler et al. (2013) reviews the how's, what's, where's, and why's of N-glycosylation for *Haloferax volcanii* in response to salinity fluctuations.

As the lead organizer of the conference, and co-editor of this eBook, it was a great pleasure for me to serve the community and orchestrate this meeting: It was a labor of love and I would gladly do it again. It was truly an unforgettable conference, everyone had a great time, and we are all looking forward to the next one, Halophiles 2016, to be held in San Juan, Puerto Rico.

### Conflict of interest statement

The author declares that the research was conducted in the absence of any commercial or financial relationships that could be construed as a potential conflict of interest.
